# Tissue Mechanics in Haired Murine Skin: Potential Implications for Skin Aging

**DOI:** 10.3389/fcell.2021.635340

**Published:** 2021-02-19

**Authors:** Hans I-Chen Harn, Chih-Chiang Chen, Sheng-Pei Wang, Mingxing Lei, Cheng-Ming Chuong

**Affiliations:** ^1^Department of Pathology, Keck School of Medicine, University of Southern California, Los Angeles, CA, United States; ^2^International Research Center of Wound Repair and Regeneration (iWRR), National Cheng Kung University, Tainan, Taiwan; ^3^Department of Dermatology, Taipei Veterans General Hospital, Taipei, Taiwan; ^4^Institute of Clinical Medicine, National Yang-Ming University, Taipei, Taiwan; ^5^Department of Dermatology, National Yang-Ming University, Taipei, Taiwan; ^6^111 Project Laboratory of Biomechanics and Tissue Repair, College of Bioengineering, Chongqing University, Chongqing, China; ^7^Key Laboratory of Biorheological Science and Technology of the Ministry of Education, College of Bioengineering, Chongqing University, Chongqing, China

**Keywords:** skin aging, extracellular matrix, macroenvironment, hair regeneration, skin tension, tissue mechanics, tensional homeostasis, microenvironment

## Abstract

During aging, the skin undergoes changes in architecture and composition. Skin aging phenotypes occur due to accumulated changes in the genome/epigenome, cytokine/cell adhesion, cell distribution/extracellular matrix (ECM), etc. Here we review data suggesting that tissue mechanics also plays a role in skin aging. While mouse and human skin share some similarities, their skin architectures differ in some respects. However, we use recent research in haired murine skin because of the available experimental data. Skin suffers from changes in both its appendages and inter-appendage regions. The elderly exhibit wrinkles and loose dermis and are more likely to suffer from wounds and superficial abrasions with poor healing. They also have a reduction in the number of skin appendages. While telogen is prolonged in aging murine skin, hair follicle stem cells can be rejuvenated to enter anagen if transplanted to a young skin environment. We highlight recent single-cell analyses performed on epidermis and aging human skin which identified new basal cell subpopulations that shift in response to wounding. This may be due to alterations of basement membrane stiffness which would change tissue mechanics in aging skin, leading to altered homeostatic dynamics. We propose that the extracellular matrix (ECM) may play a key role as a chemo-mechanical integrator of the multi-layered senescence-associated signaling pathways, dictating the tissue mechanical landscape of niche microenvironments in aging phenotypes. We show examples where failed chemo-mechanical signaling leads to deteriorating homeostasis during skin aging and suggest potential therapeutic strategies to guide future research to delay the aging processes.

## Introduction

As skin ages, it undergoes physiological and pathological changes causing a decline in both structure and function (Gilchrest, 1989; Tobin, 2017). These changes affect many aspects of skin biology, including (1) compromised barrier function and mechanical protection, (2) dampened immune responses, (3) impaired thermoregulation, (4) decreased sweat and sebum production, (5) delayed hair cycling (Chueh et al., 2013; Chen et al., 2016), and (6) delayed wound healing (Gosain and DiPietro, 2004). Aging is complex involving both intrinsic and extrinsic factors. Earlier work by Hayflick suggests cells have a fixed cellular lifespan, leading to the identification of roles of telomere in aging (Kameda et al., 2020). Recent epigenetic studies identified unique age-related methylome changes associated with the aging epigenome (Zhang et al., 2020). Environmental stresses such as somatic mutations, free radical damage, senescence-associated secretory phenotype, autophagy, chronic inflammation, etc. can also promote senescence (Di Micco et al., 2020; Tabibzadeh, 2021). These findings have identified new concepts and therapeutic targets fostering the hope that aging can be delayed and perhaps tissues can be rejuvenated by modifying the epigenome (Guarasci et al., 2019) or by activating resident tissue stem cells (Fuchs and Blau, 2020). While these are important aspects of the aging process, in this mini-review, we discuss the role of tissue mechanics in the aging skin. Tensional homeostasis, a process that balances the extracellular forces exerted on cells by extracellular matrix (ECM) or neighboring cells and the reciprocal forces, is essential to maintain cell function and tissue remodeling. While we consider this concept has a role in both human and mouse aging skin, we will focus more on the haired murine skin because of the available reports. During mouse skin aging, the tissue mechanics landscape shifts greatly, as skin homeostasis declines. Based on these reports, we suggest some potential therapeutic strategies at the end.

## The Aging Skin

Manifestations of the aging skin include rough skin textures, wrinkles, laxity, atrophy, pigmentary changes, loss of underlying fat, dry and itchy skin, inability to perspire sufficiently, hair graying, hair loss, thinning of nail plates amongst others (Farage et al., 2013). Recent reports show these traits are affected by both intrinsic (chronological) and extrinsic (non-genetic) factors (Parsons, 1996; Gunn et al., 2009; Tobin, 2017).

### Human Skin Aging and Related Diseases

Intrinsically aged skin appears dry and pale with fine wrinkles and increased laxity. This results from gradual physiologic changes include reductions of cell number, collagen production, blood flow, lipids, and rete ridges (Montagna et al., 1989; Tobin, 2017). Extrinsic human skin aging entails changes caused by environmental factors, including ultraviolet light (UV). UV damage upregulates matrix metalloproteases (MMP) (Fisher et al., 1996; Quan et al., 2013) and impacts collagen degradation and synthesis (Varani et al., 2006), as well as the production of elastotic materials in the skin. This creates a microenvironment of fragmented collagen and leads to aberrant collagen homeostasis. There is controversy regarding the effect of aging, whether intrinsic or extrinsic, on epidermal thickness (El-Domyati et al., 2002). Some studies have suggested that intrinsic aging tends to cause a slight overall thinning of the viable epidermis (Lavker, 1979; Lavker and Kligman, 1988; Branchet et al., 1990), while others have found that extrinsic aging tends to cause irregular thickening of the epidermis (Gilchrest, 1989). Recently, single-cell RNA analyses revealed a progressive accumulation of photoaging-related changes and increased chronic inflammation in aging human eyelid skin (Zou et al., 2020). Hair follicular stem cells (HFSC) were shown to be nearly normal in human androgenetic alopecia; the problem is the failure of the niche to convert bulge stem cells (SC) into hair germs, hence resulting in prolonged telogen (Garza et al., 2011). These changes also contribute to hair follicle miniaturization and lowered hair density (Matsumura et al., 2016) and the competition of HFSC fates (Liu et al., 2019).

Solar elastosis, characterized by the replacement of normal elastic fibers with a disordered mass of elastotic material near the dermal-epidermal junction, is the hallmark of photodamaged dermis (Yaar and Gilchrest, 2007). Senile purpura, characterized by recurrent formation of ecchymoses on the sun-exposed extensor surfaces of the extremities, is another disease related to reduction of connective tissue in the dermis. Age-related skin thinning and sun-induced damage of the dermal connective tissue results in inadequate support, increased fragility and rupture of the microvasculature (Fenske and Lober, 1986) as well as decreased wound tensile strength (Thomas, 2001); its cosmetic concerns can negatively impact a patient’s quality of life (McKnight et al., 2015).

### Structural and Functional Changes in Aging Skin

**FIGURE 1:**
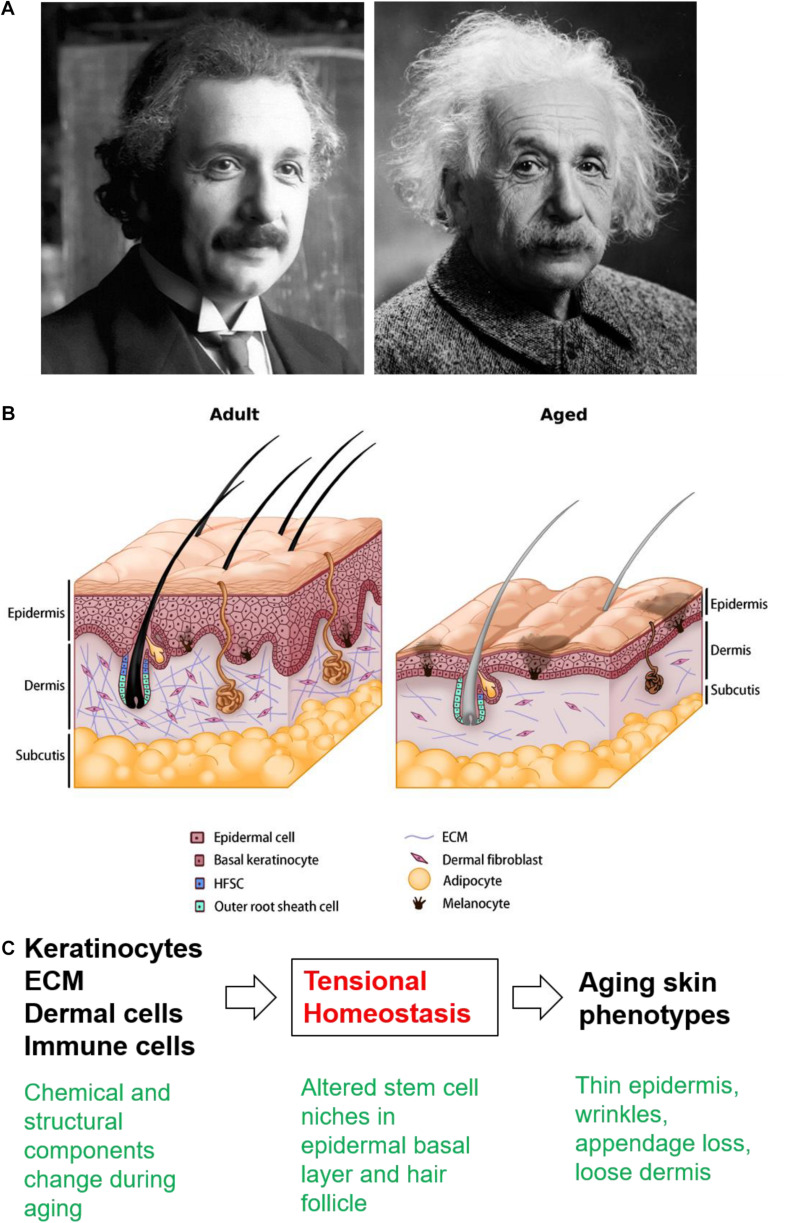
The aging skin. **(A)** Young and old Albert Einstein used to illustrate the changes in the appearance of aging skin. Source: Pixabay. **(B)** Schematic drawing showing skin architecture in the adult and aged skin. In aged skin, the overall thickness decreases progressively across all layers, accompanied by wrinkles, pigmentary changes, loss of underlying fat, hair graying, hair loss, and decreased sebaceous gland function. The reduced number and altered activity of fibroblasts have been implicated as the underlying cause of decreased dermal ECM synthesis, leading to deranged ECM support and affects collagen fibril crosslinking. The changes in decreasing cell numbers and altered ECM production and assembly also contribute to hair follicle miniaturization and lowered hair density in aged skin. **(C)** Cellular/molecular events reviewed in the text present potential new therapeutic targets.

Within the aging mouse skin, the cell number and turnover rate decrease across all cell types (Farage et al., 2013), which impacts epidermal thickness and barrier integrity (Minematsu et al., 2011). Langerhans cells also decrease in number, leading to impaired immune responses (Wulf et al., 2004). Melanocytes lose their enzyme activity, resulting in uneven pigmentation (Nishimura et al., 2005). The flattening of the dermo-epidermal junction alters the epidermal anchoring system (Le Varlet et al., 1998), and hence the skin becomes more vulnerable to dermo-epidermal separation. In the aging dermis, the cellularity, vascularity, innervation, and ECM content also decrease (Makrantonaki and Zouboulis, 2007). The structure of sweat glands becomes distorted and the number of functional sweat glands decreases (Fenske and Lober, 1986). The loss of adipose tissue hinders thermoregulatory function and other signaling roles (Zwick et al., 2018). Interestingly, aging-associated decreases in dermal thickness corresponds with increases in dermal white adipose tissue (dWAT) thickness (Kruglikov et al., 2019b; Zhang et al., 2021). Mature dermal adipocytes de-differentiate and release free fatty acids into the surrounding ECM, which further changes the metabolism and strongly modulates the physiology of adjacent dermal fibroblasts, significantly influencing their collagen expression (Kruglikov and Scherer, 2016). The reduced number and activity of fibroblasts were implicated as the cause of decreased dermal ECM synthesis, leading to deranged ECM support and adequate functions for the skin and skin appendages (Cole et al., 2018). Low fibroblast activity also affects collagen fibril crosslinking (Yang et al., 2008), resulting in a rigid but weakened dermis (Silver et al., 2003). Disorganized ECM also decreases the amount of mechanical loading experienced by the dermal fibroblasts and a possibly diminished ability of the skin to respond to mechanical stimuli, creating a vicious cycle that further deteriorates the homeostasis and physiological functioning of the aged skin (Wu et al., 2011). The structural changes in aging skin and the multilayer control of skin homeostasis (Figures 1A,B), wound healing, and HFSC interaction with the surrounding ECM will be further discussed.

### Factors Underlying How Aging Changes the Murine Skin

Adult skin SC are vital for replacing cells in tissues, but their capacity declines with age. SC are regulated by both macroenvironmental signals (Chen and Chuong, 2012; Chen et al., 2016) and their microenvironmental niche (Ge et al., 2020). On the macroenvironment level, the hair cycle in aged mouse skin shows higher and longer expression of HFSC activation inhibitors such as BMPs, DKK1, and SFRPs in the dermis (Chen et al., 2014). On the cellular level, HFSC from aged skin show defects in Nfatc1 (Keyes et al., 2013) and FoxC1 (Lay et al., 2016; Wang et al., 2016). Microenvironmental factors including ECM (Ge et al., 2020), immune, sensory nerves and arrector pili (Shwartz et al., 2020) all take part in regulating HFSC activity. Expression and maintenance of the hemidesmosome component Col17A1 are critical for skin homeostasis and skin epithelial stem cell retention (Matsumura et al., 2016; Liu et al., 2019). Tenascin C (Tnc), an element of the ECM, is shown to promote ECM integrity and is reduced in aged skin (Choi et al., 2020). Thus, both intrinsic and extrinsic factors play a role in the aging of HFSC (Lei and Chuong, 2016).

## Tensional Homeostasis: Balanced Tissue Mechanics

Tissue mechanics (Kenedi et al., 1975; Hsu et al., 2018a) can provide a new approach in contemplating stem cell regeneration in the aged skin.

### ECM/MMP and Tissue Mechanics in the Skin

The skin cells interact dynamically with the basement membrane (BM), ECM, and the dermis to shape the structure and function of the skin (Fiore et al., 2020). The dermis contains the greatest volume of ECM, which interacts with the dermal cells that generate contractile forces, especially in face of injury (Hsu et al., 2018a; Harn et al., 2019). The amount, content, and organization of ECM being synthesized and remodeled by the MMPs in the dermis is in an intricate balance to maintain the homeostasis of the skin and in aging (Naylor et al., 2011; Tracy et al., 2016). In turn, the ECM provides not only biochemical but also mechanical cues to impact cell behavior (Watt and Fujiwara, 2011). Cells sense mechanical stimuli via mechanosensors (e.g., integrin, hemidesmosomes, adherent junctions, stretch-sensitive ion channels) and translocate these signals into the nucleus for transcriptional response in the mechanotransduction process (Wong et al., 2011). Tensional homeostasis, a concept introduced by Weaver and colleagues to describe the balance between the extracellular forces exerted on the cells by neighboring cells, ECM and reciprocal forces generated by the cells (Paszek et al., 2005), is also essential for maintaining cell function and tissue remodeling in the skin (Harn et al., 2019).

### Heterogeneity of the Basal Layer and HF Niche

Recent single-cell analyses identify new sub-populations of basal cells that specifically localize at the top or bottom of rete ridges (Wang et al., 2020), suggesting a mechanical control. Interfollicular epithelial cell differentiation is best described as a single step gradualistic process with a large number of transition cells between the basal and spinous layer (Lin et al., 2020). Their homeostatic states also shift in response to wounding into four different cell states that govern cell proliferation or differentiation (Haensel et al., 2020). We speculate this flexibility of cell states shown during homeostasis is also reflected on cell mechanics; the dynamic changes of cell states also create physical changes to cellular forces that drive cell migration and collective morphological changes.

Rete ridge reductions and tissue mechanical landscape changes in aging skin can either be the cause or consequence of changes in these basal layer sub-populations. Additionally, the basement membrane (BM) adjacent to HFSC serves as an arrector pili muscle (APM) niche (Fujiwara et al., 2011), which together with the sympathetic nerve can form a dual-component niche to modulate HFSC activity (Shwartz et al., 2020). ECM dysregulation that accumulates during skin aging could also affect BM properties chemically and mechanically, which in turn alters APM homeostasis and HFSC regulation.

### Tissue Mechanics in Aging Skin: ECM Dysregulation, Wrinkling, and Wound Healing

The changes in ECM synthesis, assembly and dampened cellular response to mechanical stimuli in aged skin lead to low proliferation, discontinued migration, prolonged cell-cycle arrest, and eventually cell death (Sherratt, 2009; Wu et al., 2011). Kruglikov and Scherer reported wrinkling as a mechanical phenomenon where the mismatch of mechanical modules between adjacent epidermal-dermal or dermal-subcutaneous junctions, leads to structural instabilities inside the skin (Kruglikov and Scherer, 2018). We view wrinkles as an excessively stiff apical surface area (stratified epidermis) with a decreased basal dimension and resistance (reduced ECM, dermal cell, adipose tissue) that leads to tissue folding (Varner and Nelson, 2014). In multilayered epithelial tissue, softening, and enhanced remodeling of the BM promote tumor budding, while stiffening of the BM promotes tissue folding (Fiore et al., 2020). In parallel, what factors could contribute to the reduced rete ridges and increased wrinkles in aging skin? We propose 3 possible scenarios: (1) BM stiffness increases in aging skin, (2) loss of Col17A1 causes an increase in the ratio of differentiated cells to basal progenitor cells, and (3) decreases in dermis stiffness and length force the attached apical epidermis to bend and fold upward. The precise mechanical changes of these constituents in aging skin remains to be investigated.

Wound healing in a healthy, aged individual is delayed but not defective (Gerstein et al., 1993). This is due to their dampened cellular activity and asynchronization of pro and anti-inflammatory responses to injury (Ashcroft et al., 2002). The reduced number, activity, and migration of keratinocytes and fibroblasts in aged skin lead to delayed re-epithelialization, wound closure, and tissue reformation (Moulin et al., 2000; Ashcroft et al., 2002). On the other hand, although aged skin shows little capacity to regenerate, it is less prone to form hypertrophic scars and keloids (Hsu et al., 2018b), whose etiology is mechanically sensitive (Harn et al., 2015, 2016). Aging skin is characterized by reduced ECM deposition brought about by (1) low skin tension, (2) dampened cellular responses to mechanical stimuli coupled with (3) low pro-inflammatory signaling, and (4) the reduced capacity of dermal adipocytes to transdifferentiate into myofibroblasts (Varani et al., 2006; Zhang et al., 2019) which could underlie reduced scarring after wounding.

## Impacts With Perturbed Tensional Homeostasis

Perturbation to tissue mechanics or disruption of tensional homeostasis impacts physiological functioning of the skin, including hair cycling and wound healing (Hsu et al., 2018a; Harn et al., 2019).

### Skin Tension and Scarring vs. Regeneration

Generally, increasing skin tension or stiffness leads to higher cell activity and ECM synthesis. Molecules responsible for mechanotransduction are also important in regulating tensional homeostasis. Epidermal-specific deletion of integrin or focal adhesion kinase (FAK) results in disfigured hair follicles and dysregulated hair cycle propagation (Essayem et al., 2006). Activation of TRPV1, a transient receptor potential cation channel involved in mechano-transduction, inhibits hair shaft lengthening and induces premature catagen (Biro et al., 2006). Tension across the wound has been demonstrated as a key inducer of scarring in hypertrophic scars and keloids (Harn et al., 2019). FAK and integrins could be important mediators of these mechanotransduction processes (Wong et al., 2012), although the reduced capacity of dermal adipocytes to de- and transdifferentiate into myofibroblasts could also be an underlying factor (Zhang et al., 2019). In contrast, the African Spiny mice skin expresses a high collagen III to I ratio producing an exceptionally soft substrate (Seifert et al., 2012). Spiny mice can regenerate their skin and all of their appendages after full-thickness wounding (Jiang et al., 2019).

### Cyclic Stretch of the Dorsal Murine Skin Activates HFSC

The impact of skin “loosening” during aging can be deduced from the effects of mechanical force on the homeostasis of HFSC. The amount and duration of skin mechanical stretching result in HFSC proliferation (Chu et al., 2019). This depends on three major components. First, the counterbalance between Wnt and BMP-2; second, M2 subtype macrophage polarization; third, growth factor secretion, especially HGF and IGF-1 (Chu et al., 2019). However, this mechanical activation of hair cells seems compromised in the stiff and less extendable aged mice skin (Lynch et al., 2017; Meador et al., 2020) which has difficulty in eliciting a desirable regeneration response via stretching. How mechanical force, ECM and immune cells contribute to aging skin’s inadequate regenerative activity remains to be explored.

### Hair Plucking in the Dorsal Murine Skin Triggers a Regenerative Immune Response

External mechanical forces such as hair plucking can also trigger the immune response-mediated regenerative process. Properly arranged hair plucking can trigger an efficient regenerative response mediated through a two-step immune response cascade, called quorum sensing (Chen et al., 2015). These examples demonstrate the importance of tissue mechanics on maintaining homeostasis of the skin and its response to injury.

## Recent Discoveries for Therapeutic Strategies Against Aging Skin

Although currently there is no prominent strategy for developing new drugs against skin aging, there are a few chemical and mechanical approaches that have demonstrated potential.

### Chemical-Based Approach: PRP, Follistatin, HES1, ADSC, and dWAT

Platelet-rich plasma (PRP) has been adopted to treat many degenerative disorders including hair loss (Li et al., 2012; Khatu et al., 2014) with reasonable success despite variations in current protocols (Ayatollahi et al., 2017; Picard et al., 2017). In principle, this approach is based on the various growth factors released (Gentile et al., 2017); however, its precise mechanism has not been elucidated. Charles-de-Sa et al. demonstrated that dWAT completely disappears after PRP injection and is replaced by fibrotic tissue, which is very similar to the effect observed after Bleomycin injection (Charles-de-Sa et al., 2018).

Replenishing effective growth factors or activating specific downstream genes such as Wnt-follistatin and follistatin-like 1 protein have been tested as a treatment for alopecia (Zimber et al., 2011; Chen et al., 2014). Pharmacological activation of HES1 alleviated the cellular senescence of aged dermal fibroblasts (Zou et al., 2020). Adipose-derived stem cells (ADSC) have shown effects related to dermal fibroblast activation via secretion of growth factors (Kim et al., 2011). Subcutaneous injection of ADSC significantly increased collagen density, dermal thickness, and fibroblast number in hairless mice (Fukuoka et al., 2017), and also stimulated collagen synthesis of human dermal fibroblasts (Kim et al., 2009).

The adipocytes in the dWAT have high plasticity and undergo reversible dedifferentiation, in which adipocyte-derived preadipocytes trans-differentiate into myoblasts and are involved not only in the physiological hair cycle but also under pathological conditions such as hypertrophic scarring, systemic sclerosis and androgenetic alopecia (Zhang et al., 2019). During proliferation, differentiation and dedifferentiation, various extracellular vesicles are secreted to the nearby HF and in turn stimulate its hair cycle (Kruglikov et al., 2019b). Lastly, Caveolin-1 (Cav-1) is upregulated in aged skin and is associated with altered dermal thickness, dWAT expansion and ECM expression, which sequentially influences the mechanical properties of the skin layers (Kruglikov et al., 2019a,b). This makes Cav-1 a potential candidate for anti-aging treatment especially since Mevastatin has been shown to target Cav-1 as an effective treatment for chronic wound healing (Sawaya et al., 2019).

### Mechanical Approach: Stretching and ECM/MMP Perturbation

Although the effectiveness of stretching-induced HF activation is less pronounced in aging mice (Chu et al., 2019), interestingly, subcutaneous injection of cross-linked hyaluronic acid stimulates collagen synthesis and partially restores dermal ECM that are lost in photodamaged skin (Bukhari et al., 2018; Yamada and Prow, 2020). It is speculated that this effect is achieved by mechanical stretching of the dermis and the activation of dermal fibroblasts (Chu et al., 2019). It also was demonstrated that cross-linked HA can significantly influence dWAT and subcutaneous WAT which modifies the dermal-subcutaneous junction (Kruglikov and Wollina, 2015), and directly but differentially affects the mature adipocytes and preadipocytes (Nadra et al., unpublished). Other studies have also focused on securing collagen integrity via suppressing MMP production and activity (Oh et al., 2020a), particularly in alleviating UVA-mediated suppression of collagen expression by stimulating TGF-β/Smad signaling (Oh et al., 2020b).

## Summary

We review the physiological changes associated with skin aging (Table 1 and Figure 1C) and highlight the potential role of tensional homeostasis. Utilizing and providing a chemically or mechanically induced young environment might rescue aging phenotypes in the skin. This approach could potentially rescue basal layer homeostasis to control (1) epidermal thickness, (2) wound healing, (3) activation of HFSC, and (4) cell reprogramming during wound-induced hair neogenesis (Bhoopalam et al., 2020). While the haired murine skin provides an experimental model that reveals new molecular understanding summarized here, aging human skin has features that do not exist in the mouse model and await further exploration.

**TABLE 1:** Comparison of key differences between young and aged skin.

**Category**	**Young skin**	**Aged skin**	**References**
Structural features	Moist, smooth skin surface. Rete ridges, and sufficient subcutaneous fat, normal perspire function. Normal hair density and pigmentation.	Decrease thickness, dry, fine wrinkles, pigmentary changes. Reduced rete ridges and underlying fat, inability to perspire sufficiently, hair graying, hair loss, decrease sebaceous gland function, thinning of nail plates.	Farage et al., 2013; Tobin, 2017
Macroenvironment	Regulated hair cycle, homeostasis, normal physiological function, ECM and MMP balance, regulated expression of inhibitory and activating factors. Normal wound healing.	Prolonged telogen, deteriorated homeostasis and physiological function. Higher expression of MMPs. Longer expression of BMPs, DKK1, SFRP, downregulation of follistatin. Delayed wound healing. Somatic mutation, free radical damage, senescence-associated secretory phenotype, autophagy, chronic inflammation.	Chen et al., 2014; Fuchs and Blau, 2020; Kameda et al., 2020
Microenvironment	Normal HFSC and dermal cell number and function. Low DNA damage. Normal Nfatc1, FoxC1 expression. Normal and proper ECM production and crosslinking. COL17A1 maintenance. High Tnc expression.	Reduced HFSC and dermal cell number. Accumulated DNA damage. Defect Nfatc1 and FoxC1 expression. Reduced ECM production and deranged crosslinking. Increased COL17A1 proteolysis. Reduced Tnc expression. Aging epigenome.	Keyes et al., 2013; Lay et al., 2016; Matsumura et al., 2016; Wang et al., 2016; Guarasci et al., 2019; Liu et al., 2019; Choi et al., 2020; Di Micco et al., 2020; Zhang et al., 2020; Tabibzadeh, 2021
Tissue mechanics	Prone to hypertrophic scar and keloid. Activation of TRPV1 induces premature catagen. Cyclic stretch induces anagen re-entry. Hair plucking triggers a regenerative immune response.	Rigid dermis, less resistant to shearing force. Dampened cellular activity to mechanical stimuli. Less prone to scaring.	Chen et al., 2015; Chu et al., 2019; Harn et al., 2019

*Summary of changes during the aging of haired murine skin, the micro- and macroenvironmental signals contributing to HFSC maintenance.*

## Data Availability Statement

The original contributions presented in the study are included in the article/supplementary material, further inquiries can be directed to the corresponding author/s.

## Ethics Statement

Written informed consent was not obtained from the individual(s) for the publication of any potentially identifiable images or data included in this article.

## Author Contributions

CCC and SPW contributed more to the clinical aspect. HICH, ML, and CMC contributed more to the basic research elements. All authors contributed to the writing, organization, and editing of the manuscript. The manuscript was proofread by Dr. R. B. Widelitz.

## Conflict of Interest

The authors declare that the research was conducted in the absence of any commercial or financial relationships that could be construed as a potential conflict of interest.
